# COMPOSITE MEASURES FOR MULTIDIMENSIONAL SOCIAL EXCLUSION: AN APPLICATION TO THE IRISH LONGITUDINAL STUDY ON AGEING

**DOI:** 10.1093/geroni/igz038.2954

**Published:** 2019-11-08

**Authors:** Sinead Keogh, Stephen O’ Neill, Kieran Walsh

**Affiliations:** 1 National University of Ireland Galway, Galway, Ireland, Ireland; 2 NUI Galway, Galway, Ireland, Ireland

## Abstract

The measurement of the complex, multidimensional and dynamic concept of old-age social exclusion has been constrained due to theoretical and methodological challenges as well as a reliance on secondary data sources not designed to collect social exclusion indicators. Limitations in measuring social exclusion in later life hinder the expansion of our empirical and conceptual understanding of social exclusion. In this paper, we seek to address these limitations by developing a composite measure of old-age social exclusion using three methods: 1) normalisation through re-scaling with linear aggregation, 2) a sum-of-scores approach with an applied threshold and, 3) classification and regression trees (CART), a machine learning approach. Using the conceptual framework of old-age exclusion presented by Walsh et al., (2017), these three approaches are applied empirically with data from Wave 1 of The Irish Longitudinal Study on Ageing (TILDA). The measures are assessed in terms of their ability to explain a validated measure of psychological well-being. Results suggest that despite the challenges associated with secondary data and measurement techniques that implicitly measure social exclusion, the newly proposed composite measure computed using CART performed better than the other two measures which are more prevalent in the literature.

